# A Neural-Network-Based Watermarking Method Approximating JPEG Quantization

**DOI:** 10.3390/jimaging10060138

**Published:** 2024-06-06

**Authors:** Shingo Yamauchi, Masaki Kawamura

**Affiliations:** Graduate School of Sciences and Technology for Innovation, Yamaguchi University, Yamaguchi 753-8512, Japan; c028vbw@yamaguchi-u.ac.jp

**Keywords:** watermarking method, neural network, activation function, JPEG compression

## Abstract

We propose a neural-network-based watermarking method that introduces the quantized activation function that approximates the quantization of JPEG compression. Many neural-network-based watermarking methods have been proposed. Conventional methods have acquired robustness against various attacks by introducing an attack simulation layer between the embedding network and the extraction network. The quantization process of JPEG compression is replaced by the noise addition process in the attack layer of conventional methods. In this paper, we propose a quantized activation function that can simulate the JPEG quantization standard as it is in order to improve the robustness against the JPEG compression. Our quantized activation function consists of several hyperbolic tangent functions and is applied as an activation function for neural networks. Our network was introduced in the attack layer of ReDMark proposed by Ahmadi et al. to compare it with their method. That is, the embedding and extraction networks had the same structure. We compared the usual JPEG compressed images and the images applying the quantized activation function. The results showed that a network with quantized activation functions can approximate JPEG compression with high accuracy. We also compared the bit error rate (BER) of estimated watermarks generated by our network with those generated by ReDMark. We found that our network was able to produce estimated watermarks with lower BERs than those of ReDMark. Therefore, our network outperformed the conventional method with respect to image quality and BER.

## 1. Introduction

People are now easily able to upload photos and illustrations to the Internet, owing to smartphones and personal computers. To protect content creators, we need to prevent unauthorized copying and other abuses because digital content is not degraded by copying or transmissions. Digital watermarking is effective against such unauthorized use.

In digital watermarking, secret information is embedded in digital content by making slight changes to the content. In the case of an image, the image in which the information is embedded is called a stego-image, and the embedded information is called a digital watermark. There are two types of digital watermarking: blind and non-blind. The blind method does not require the original image to extract the watermark from the stego-image. However, the non-blind method requires the original image when extracting a watermark from a stego-image. Therefore, the blind method is more practical. In addition, because stego-images may be attacked by various kinds of image processing, watermarking methods must have the ability to extract watermarks from degraded stego-images. Two types of attacks on stego-images can occur: geometric attacks such as rotation, scaling, and cropping, and non-geometric attacks such as noise addition and JPEG compression [1].

Neural-network-based methods have been proposed. In single-stage training, where the embedding and extraction are performed in a single network, the network has been trained to output a watermark from an input image [2,3]. The overall performance of the network is low because the relationship between the image and the watermark is trained individually. To improve performance, watermarking methods using autoencoders (AE) have been proposed [4,5,6]. The input layer to the middle layer is called the embedding network, and the middle to the output layer is called the extraction network. Both the original image and the watermark are input into the input layer of the AE, and the identity mapping is learned to retrieve them in the output layer. The stego-image is extracted from the middle layer [6]. Since the original image is unnecessary during extraction, it is often omitted to output only the watermark. Furthermore, AE with convolutional neural networks has been proposed [7]. An adversarial network has also been added to improve image quality [8]. DARI-Mark [9] is a DNN-based watermarking method using attention to determine the embedding regions. It can find non-significant regions that are insensitive to the human eye and increases robustness by embedding the watermark with larger intensities. Thus, end-to-end models were proposed [6,7,8,9,10,11]. However, a huge training dataset was needed to train the connections as the network became more complex. Although data augmentation was sometimes introduced, a model with internal networks mimicking attacks was proposed in order to train on a relatively small training dataset [8,10,11].

HiDDeN [8], proposed by Zhu et al., has an attack layer that simulates attacks such as Gaussian blur, per-pixel dropout, cropping, and JPEG compression attacks on images during training. Here, the implementation of JPEG compression is approximated by JPEG-Mask, which sets the high-frequency components of the discrete cosine transform (DCT) coefficients to zero, and JPEG-Drop, which uses progressive dropout to eliminate the high-frequency components of the DCT coefficients. Therefore, this implementation does not meet the standard for quantization in the JPEG compression process. It has also been noted that the JPEG-Mask and JPEG-Drop layers of HiDDeN do not provide sufficient performance for the robustness of the JPEG compression [12,13]. JPEGdiff is a method of approximating around the quantized values in JPEG compression by a cubic function. Hamamoto and Kawamura’s method [10] also introduces a layer of additive white Gaussian noise as an attack layer to improve robustness against JPEG compression. Moreover, ReDMark proposed by Ahmadi et al. [11] has attack layers implementing salt-and-pepper noise, Gaussian noise, JPEG compression, and mean smoothing filters. The quantization of the JPEG compression is approximated by adding uniform noise. As described, the quantization process has been replaced by the process of adding noise, and the quantization process as per the JPEG standard has not been introduced.

Adversarial samples are a problem in the field of pattern recognition. They are generated by adding distortions to images to misclassify them. To avoid misclassification, a pattern recognition method using JPEG compressed images has been proposed [14]. JPEG compression is expected to effectively reduce noise while preserving the information needed for pattern recognition. However, JPEGdiff has been proposed as a way to break this technique [12]. By approximating the JPEG quantization with a differentiable function, a JPEG-resistant adversarial image can be generated. Therefore, approximating the JPEG quantization with a smooth function may affect the performance of the model.

In our previous work [15], we proposed a quantization activation function (QAF) that can simulate the quantization of JPEG compression according to a standard. That model consists of a network that introduces the QAF into the AE-based model proposed by Hamamoto and Kawamura [6]. Better performance was obtained in terms of JPEG compression robustness than the AE-based model [6]. The effectiveness of the QAF has been demonstrated in our previous work. However, in that model, a QAF with a constant quantization width was used instead of the quantization table. In this paper, we apply the QAF to the attack layer of the ReDMark [11], which is a CNN-based model, rather than an AE-based model. Furthermore, the proposed method uses the quantization table-based QAF. The robustness against the JPEG compression is expected to be improved using the QAF. The effectiveness of our method is evaluated by comparing JPEG-compressed images with QAF-applied images. The image quality of the stego-image is also evaluated.

The rest of the paper is organized as follows. In Section 2, the process of JPEG quantization is explained. In Section 3, we describe the ReDMark and, in addition, we address our previous work. In Section 4, we define the quantized activation function and describe the structure of the proposed network. In Section 5, we show the effectiveness of the function and demonstrate the performance of our network in computer simulations. The last section concludes the paper.

## 2. Preliminary: JPEG Quantization

**Figure 1:**
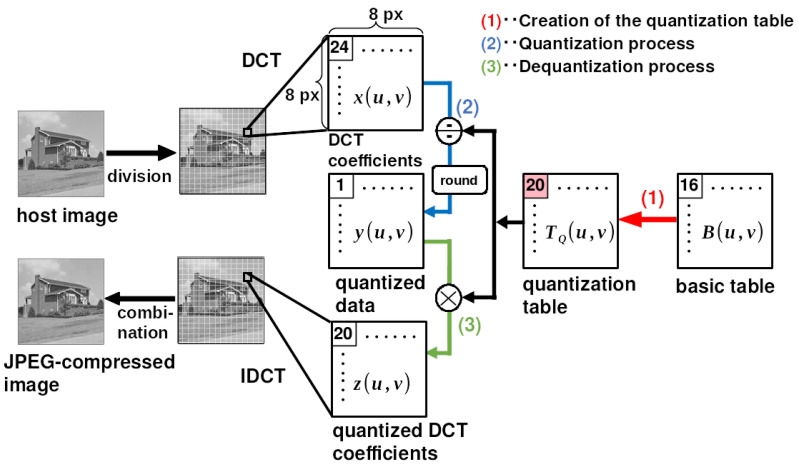
JPEG compression quantization for luminance components. (1) Creation of the quantization table $T_{Q}$, (2) the quantization process, and (3) the dequantization process.

JPEG compression is a lossy compression that reduces the amount of information in an image to reduce the file size. In this kind of compression, an image is divided into 8×8-pixel blocks. Then, in each block, the processes of the DCT, quantization, and entropy coding are sequentially performed. In JPEG compression, the process of reducing the amount of information is the quantization process of the DCT coefficients. We focus on the quantization of the DCT coefficients of the luminance component in an image because the watermark is embedded in these coefficients of the image. Figure 1 shows the quantization process in JPEG compression for DCT coefficients [16]. The process consists of three steps: (1) the creation of the quantization table $T_{Q}$, (2) the quantization process, and (3) the dequantization process.

During the quantization process, the DCT coefficients are quantized based on a default basic table or a self-defined basic table. The default basic table B is defined as

(1) $$B=\left[\begin{array}{cccccccc} 16 & 11 & 10 & 16 & 24 & 40 & 51 & 61\\ 12 & 12 & 14 & 19 & 26 & 58 & 60 & 55\\ 14 & 13 & 16 & 24 & 40 & 57 & 69 & 56\\ 14 & 17 & 22 & 29 & 51 & 87 & 80 & 62\\ 18 & 22 & 37 & 56 & 68 & 109 & 103 & 77\\ 24 & 35 & 55 & 64 & 81 & 104 & 113 & 92\\ 49 & 64 & 78 & 87 & 103 & 121 & 120 & 101\\ 72 & 92 & 95 & 98 & 112 & 100 & 103 & 99 \end{array}\right].$$

The quantization table is then determined using the quality factor (*Q*) and the basic table B. The quantization table $T_{Q}\left(u,v\right)$ for the quantization level *Q* at coordinates (u,v),u=0,1⋯,7,v=0,1⋯,7 is defined as

(2) $$T_{Q}\left(u,v\right)=\left\lfloor \frac{B(u,v)s(Q)+50}{100}\right\rfloor ,$$

where ⌊·⌋ is the floor function and where B(u,v) is the (u,v) component of the basic table B. Also, the scaling factor s(Q) is given by

(3) $$s\left(Q\right)=\left\{\begin{array}{cc} \frac{5000}{Q} & \left(Q<50\right)\\ 200-2Q & \left(Q\geq 50\right) \end{array}\right..$$

The quantization process is performed using the quantization table $T_{Q}$. Let y(u,v) be the quantized data, and let x(u,v) be the DCT coefficients in an 8×8-pixel block. The quantization process is performed as

(4) $$y\left(u,v\right)=\mathrm{round}\left(\frac{x(u,v)}{T_{Q}\left(u,v\right)}\right),$$

where

(5) $$\mathrm{round}\left(a\right)=\left\{\begin{array}{cc} \left\lfloor a+0.5\right\rfloor & \left(a\geq 0\right)\\ -\lfloor -a+0.5\rfloor & \left(a<0\right) \end{array}\right..$$

Let z(u,v) be the quantized DCT coefficients; then, the dequantization process is performed as

(6) $$z\left(u,v\right)=y\left(u,v\right)T_{Q}\left(u,v\right).$$

## 3. Related Works

### 3.1. ReDMark

**Figure 2:**
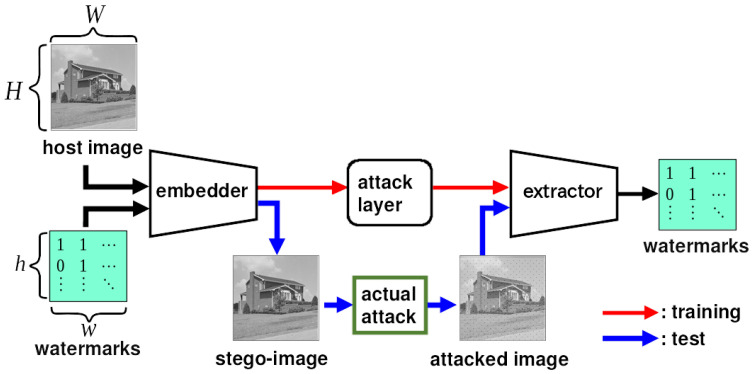
Overall structure of ReDMark [11]. The red and blue arrows represent the process flow during training and testing, respectively.

Figure 2 shows the overall structure of ReDMark [11], which consists of an embedding network, an extraction network, and an attack layer. The *h* and *w* are the height and width of the 2D watermark, and *H* and *W* are the height and width of the original and stego-images. The images are divided into M×N-pixel blocks, where M=N=8 as it is in ReDMark. The process flow during training is illustrated by the red arrows in Figure 2. The host image and watermark are fed to the embedding network, and the attack layer degrades the generated stego-image. By feeding the degraded image to the extraction network, the extraction network learns to extract the watermark from the degraded image. After training, the embedding and extraction networks are used individually. The process flow during testing is illustrated by the blue arrows in Figure 2. A stego-image is generated by the embedding network. This image is published and attacked. Let us assume that the attack is to compress the image by some JPEG tool. When the attacked image is obtained, the watermark is extracted from the image in the extraction network.

In ReDMark [11], normalization and reshaping are performed on the input image in preprocessing. For the input image $I_{\mathrm{in}}\left(i,j\right),i=0,1,\cdots ,H-1,j=0,1,\cdots ,W-1$, the normalized image is given by

(7) $$I\left(i,j\right)=\frac{I_{\mathrm{in}}\left(i,j\right)-128}{255}.$$

Reshape is an operation that divides an image into M×N-pixel blocks and transforms them into a 3D tensor representation. The image size H×W is assumed to satisfy H=hM,W=wN. The reshaped image is represented by a three-dimensional tensor of h×w×MN. This image is called the image tensor of size (h,w,MN). If necessary, the tensor is inverse transformed back to its original dimension.

#### 3.1.1. Embedding Network

**Figure 3:**
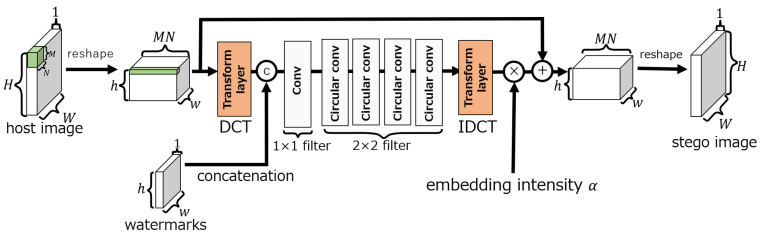
Embedding network of ReDMark [11].

**Figure 4:**
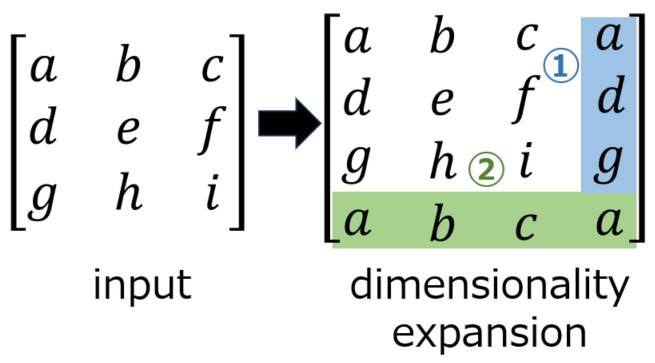
Extended input in a circular convolution layer. ➀ Extended in the column direction. ➁ Extended in the row direction.

The embedding network, as shown in Figure 3, consists of three layers: convolution, circular convolution, and transform. The transform layer can perform lossless linear transforms using 1×1 convolutional layers, e.g., the DCT, wavelet transform, and Hadamard transform. In our method, the DCT is selected as the transform layer, as it is in ReDMark. The circular convolution layer extends the input to make it cyclic before the convolution is performed. Figure 4 shows an example of applying a circular convolution layer with a 2×2 filter when the input is 3×3 pixels. When a circular convolution layer is used, the dimension of the output after convolution is the same as the dimension of the input.

In the embedding network, the convolution and circular convolution layers use 1×1 and 2×2 filters, respectively, and both have 64 filters. In each layer, an exponential linear unit (ELU) [17] activation function is used. The output of the embedding network is obtained by summing the output of the transform layer performing the inverse DCT (IDCT) (for the IDCT layer) with the input image tensor of size (h,w,MN) and then by performing the inverse process of reshaping. Here, the output of the IDCT layer can be adjusted by the embedding intensity α. The intensity is fixed as α=1 during training and can be changed during an evaluation.

#### 3.1.2. Extraction Network

**Figure 5:**
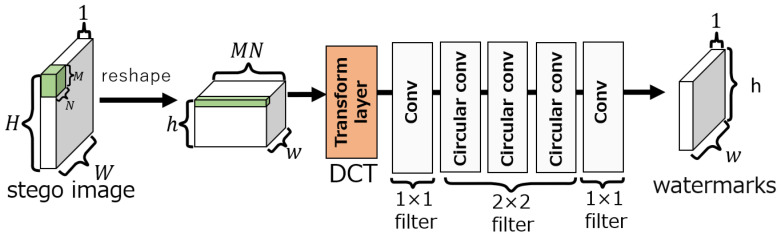
Extraction network of ReDMark [11].

The extraction network consists of a convolution layer, a circular convolution layer, and a transform layer as shown in Figure 5. The transform layer of the extraction network also performs the DCT. The filter sizes of the convolutional and circular convolutional layers are 1×1 and 2×2, respectively. The number of filters is 64 for the fourth layer and 1 for the fifth layer. The activation function up to the fourth layer is ELU [17], and a sigmoid function is used in the fifth layer. Let $p_{o}\left(i,j\right)$ be the output of the extraction network, and the estimated watermark $p_{e}\left(i,j\right)$ is given by

(8) $$p_{e}\left(i,j\right)=\left\{\begin{array}{cc} 1, & p_{o}\left(i,j\right)>0.5\\ 0, & p_{o}\left(i,j\right)\leq 0.5 \end{array}\right..$$

#### 3.1.3. Attack Layer

The attack layer lies between the embedding and the extraction networks and operates when ReDMark is trained. The attack layer itself is not trained. By simulating possible attacks on the stego-image and feeding the attacked image to the extraction network, the network can be trained to extract the watermark from the degraded image. Various attacks can be simulated in the attack layer. In the attack layer of ReDMark [11], three networks were implemented according to the type of attacks: a GT-Net (Gaussian-trained network), a JT-Net (JPEG-trained network), and a MT-Net (multi-attack-trained network).

In ReDMark, the quantization process is approximated using the quantization table $T_{Q}\left(u,v\right)$ and uniform noise ϵ. Let x(u,v) represent the DCT coefficients of an 8×8-pixel block of a stego-image, and the quantized DCT coefficients z(u,v) are given as

(9) $$\begin{array}{ccc} z(u,v) & = & \left(\frac{x(u,v)}{T_{Q}\left(u,v\right)}+\epsilon \right)T_{Q}\left(u,v\right) \end{array}$$

(10) $$\begin{array}{ccc} & = & x\left(u,v\right)+T_{Q}\left(u,v\right)\epsilon , \end{array}$$

where ϵ represents noise subject to a uniform distribution in the interval $\left[-0.5,0.5\right]$. In other words, the quantization process is equivalent to adding a uniform noise ϵ proportional to the quantization table $T_{Q}\left(u,v\right)$.

### 3.2. JPEGdiff

In the quantization of JPEG compression, the DCT coefficient values are converted to integers. This causes a problem that the activation function cannot be differentiated when training a neural network. For example, the JPEG-Mask and JPEG-Drop layers of HiDDeN do not provide sufficient performance against robustness for JPEG compression. Therefore, the JPEGdiff, which approximates the activation function to a cubic function around the quantized value, has been proposed [12,13]. This approximation can reduce the number of non-differentiable points and reduce the number of regions with a zero gradient. As a result, performance is improved by training. The JPEGdiff is given by

(11) $$\mathrm{JPEGdiff}\left(x\right)=\mathrm{round}\left(x\right)+{\left(x-\mathrm{round}(x)\right)}^{3}.$$

Note that this function still has non-differentiable points on the boundaries of the intervals.

### 3.3. Previous Work

In our previous work [15], we proposed a quantized activation function (QAF). This function was a functional representation of the quantization process of JPEG compression. Specifically, the function QAF(x) consists of several hyperbolic tangent functions and returns the quantized value of the argument *x*. The proposed QAF was applied to the attack layer of the AE-based model proposed by Hamamoto and Kawamura [6]. Moreover, all DCT coefficients x(u,v) were quantized with the same intensity. In other words, the value of the quantization table $T_{Q}\left(u,v\right)$ was constant as given by

(12) $$\begin{array}{ccc} T_{Q}\left(u,v\right) & = & \delta , \end{array}$$

where δ is a constant. Even with a constant quantization table, the previous method had a certain level of tolerance to JPEG compression. However, if the original values of the quantization table could be applied, the tolerance could be enhanced.

## 4. Proposed Method

We propose a quantization table-based QAF, and apply it to the attack layer of the ReDMark [11], which is a CNN-based model rather than an AE-based model. To demonstrate the effectiveness of the QAF, we compare the proposed layer using the QAF with the JT-Net of ReDMark [11]. The embedding and extraction networks of the proposed method are the same as those of ReDMark.

### 4.1. Quantized Activation Function

**Figure 6:**
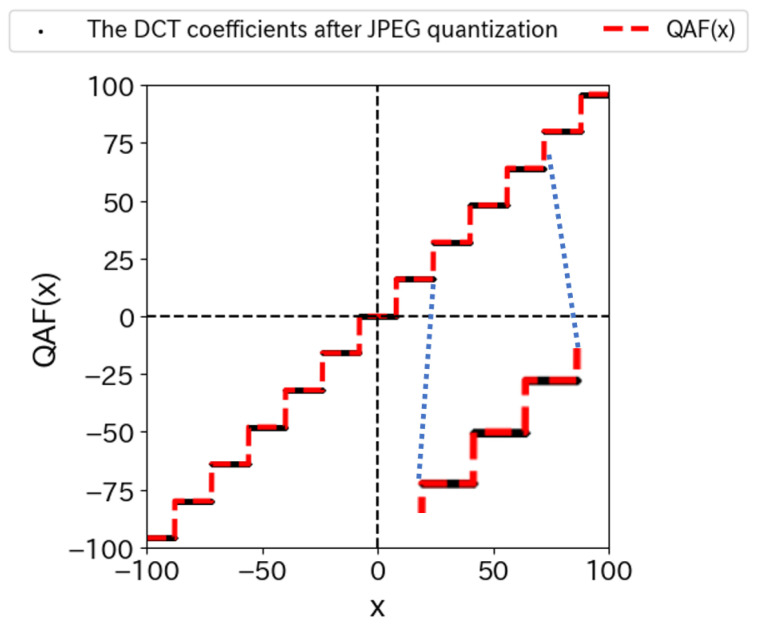
Overview of the quantized activation function: QAF(x|16) with slope β=1000 and number of hyperbolic tangent functions, n=500. The blue dotted line indicates that the function has been expanded in this range.

We propose a quantization table-based QAF for neural networks to implement the quantization process of the JPEG compression according to the standard. The QAF consists of *n* hyperbolic tangent (tanh) functions and is defined as

(13) $$\mathrm{QAF}\left(x|t_{Q}\right)=\sum_{i=0}^{n}\frac{t_{Q}}{2}\tanh\left\{\beta \left(x\pm t_{Q}\left(\frac{1}{2}+i\right)\right)\right\},$$

where $t_{Q}$ is the value of the quantization table $T_{Q}\left(u,v\right)$ when the quantization level is *Q*. The parameters *n* and β denote the number and slope of the tanh functions, respectively. The red dashed line in Figure 6 represents $\mathrm{QAF}\left(x|16\right)$ when the slope β=1000 and the value of the quantization table $T_{Q}\left(u,v\right)=16$. In addition, the values of the DCT coefficients after JPEG quantization are plotted with a black line. We can see that they are almost the same.

**Figure 7:**
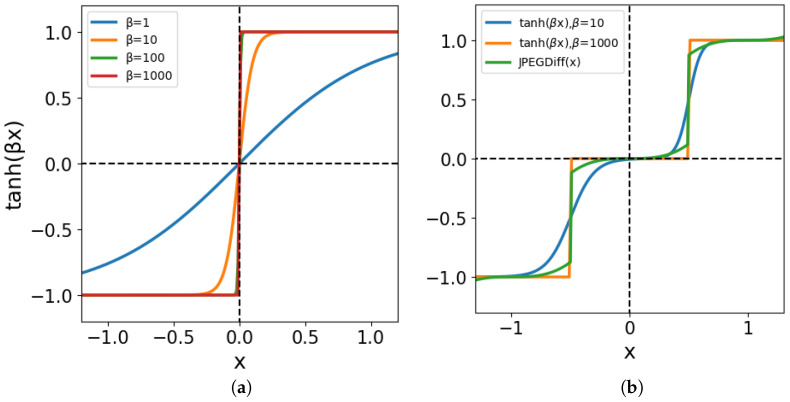
Activation functions: (**a**) the hyperbolic tangent functions for slopes β=1,10,100,1000. (**b**) JPEGdiff vs. QAF.

Quantization is essentially a rounding operation to integer values. It should therefore be represented by a discontinuous, step-like function. In other words, a sign function should be used for the representation of JPEG quantization (13). However, the tanh function was used in the proposed method. When training a neural network, the differentiable function works better for training. Therefore, we chose the tanh function, which is a continuous function. Figure 7a shows the tanh functions for different slopes β=1,10,100,1000. We can see that the slope becomes steeper and asymptotically closer to the sign function as β increases. When the slope of tanh is set to β→∞, it asymptotically approaches the sign function. For practical use, a large value of β can approximate the quantization with sufficient accuracy.

Let us see how the quantization with QAF differs from the quantization with JPEGdiff. Figure 7b shows a comparison of JPEGdiff (11) with QAF functions. The green curve represents the JPEGdiff, and the blue and orange curves represent the QAF at slopes β=10,1000. We can see that the JPEGdiff has discontinuities, while the QAF is smooth. Since the QAF has no discontinuities, the network may be better trained.

Finally, we consider the number *n* of tanh functions. The number *n* depends on the value of $T_{Q}\left(u,v\right)$. If the minimum value of $T_{Q}\left(u,v\right)=1$, because the maximum value of the DCT coefficients is 2040, then at most, 2039 tanh functions are required. A sufficiently large constant *n* is chosen because it does not matter if the possible values of the QAF exceed the maximum value of the DCT coefficients.

### 4.2. Proposed Attack Layer

**Figure 8:**
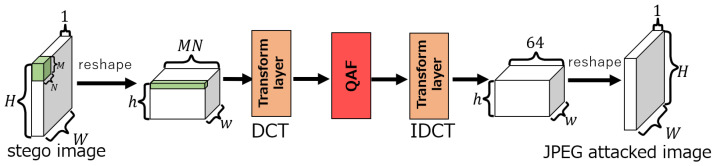
Proposed attack layer.

The proposed attack layer consists of three sublayers: a DCT layer, a layer introducing QAF (QAF layer), and an IDCT layer. Note that in our network, the DCT coefficients are quantized as in the JPEG compression. Figure 8 shows the structure of the network. The embedding and extraction networks have the same structure as that of ReDMark [11]. The output O(i,j,k),i=0,1,⋯,h−1,j=0,1,⋯,w−1,k=0,1,⋯,MN−1 of the DCT layer is processed by the QAF (13) at the QAF layer. The output z(i,j,k) of this layer is calculated by

(14) $$z\left(i,j,k\right)=\mathrm{QAF}\left(O\left(i,j,k\right)\left|\frac{T_{Q}\left(u,v\right)}{255}\right.\right).$$

Note that the quantization table $T_{Q}\left(u,v\right)$ is divided by 255 because it is normalized by (7). The quantization table values in (14) are determined according to $T_{Q}\left(u,v\right)$ because the quantization table values are different for each of the coordinates (u,v) of the DCT coefficients. The coordinates (u,v) are defined by

(15) $$u=\left\lfloor \frac{k}{8}\right\rfloor ,\;v=k-8\left\lfloor \frac{k}{8}\right\rfloor .$$

The attack layer [11] in ReDMark performs noise addition to the coefficients, while the one in our network performs the quantization with the QAF.

### 4.3. Training Method

The embedding and extraction networks are trained in the same way as they were in ReDMark [11], respectively. The loss function $L_{1}$ of the embedding network is defined as

(16) $$L_{1}=1-\mathrm{SSIM}\left(I,I_{o}\right),$$

where SSIM represents the structural similarity function (SSIM) [18], which measures the structural similarity between two images. The closer it is to 1.0, the larger the similarity between the two images [18]. It is defined in

(17) $$\mathrm{SSIM}\left(I,I_{o}\right)=\frac{\left(2\mu \mu_{o}+c_{1}\right)\left(2\mathrm{Cov}\left(I,I_{o}\right)+c_{2}\right)}{\left(\mu^{2}\mu_{o}^{2}+c_{1}\right)\left(\sigma^{2}\sigma_{o}^{2}+c_{2}\right)},$$

where I is the original image given as a teacher and where $I_{o}$ is the output of the embedding network. μ and $\mu_{o}$ are the means of I and $I_{o}$, respectively. σ and $\sigma_{o}$ are the variances of each image, and $\mathrm{Cov}(I,I_{o})$ represents the covariance between the two images. Let $c_{1}$ and $c_{2}$ be constants, and let $c_{1}=10^{-4},c_{2}=9\times 10^{-4}$. Because the output of the embedding network takes a real number, the stego-image $I_{\mathrm{st}}$ is obtained by converting it back to 256 levels of the pixel value. That is, it is given by

(18) $$I_{\mathrm{st}}\left(i,j\right)=\left\{\begin{array}{cc} 255, & I_{o}\left(i,j\right)>0.5\\ \left\lfloor 255\;I_{o}\left(i,j\right)+128\right\rfloor . & -0.5\leq I_{o}\left(i,j\right)\leq 0.5\\ \;0, & I_{o}\left(i,j\right)<-0.5 \end{array}\right..$$

The loss function $L_{2}$ of the extraction network is defined as

(19) $$\begin{array}{ccc} L_{2} & = & -\sum_{i=0}^{h-1}\sum_{j=0}^{w-1}\left\{p\left(i,j\right)\log\left(p_{o}\left(i,j\right)\right)+\left(1-p\left(i,j\right)\right)\log\left(1-p_{o}\left(i,j\right)\right)\right\}, \end{array}$$

where p is the watermark used as a teacher for the extraction network. $p_{o}$ is the output of the sigmoid function of the extraction network. That is, the value of the element takes a value between 0 and 1. The total loss function *L* of our network is defined as

(20) $$L=\gamma L_{1}+\left(1-\gamma \right)L_{2},$$

where the parameter γ determines the balance between the two loss functions. The embedding and extraction network are trained by the back propagation [19] using stochastic gradient descent (SGD) as the optimization method.

## 5. Computer Simulations

### 5.1. Evaluation of the QAF

First, the ability that the QAF function can approximate the quantization process of JPEG compression was assessed, using computer simulations. Because the DCT and quantization were applied to 8×8-pixel blocks in the JPEG compression, the QAF was also applied to 8×8-pixel blocks. The evaluation images were taken from a dataset provided by the University of Granada [20]. They consist of 49 images of 512×512 pixels. Each image was normalized by (7). The 512×512-pixel image is divided into blocks of 8×8 pixels, resulting in 64×64 blocks. These blocks were indexed in raster scan order as μ=1,2,3,⋯,4096. The DCT was performed on each block. Let $I_{b}^{\mu }\left(u,v\right),\mu =1,2,3,\cdots ,4096$ be the DCT coefficients of the μ-th block. The QAF was applied to the μ-th block as

(21) $$I_{\mathrm{QAF}}^{\mu }\left(i,j\right)=\mathrm{QAF}\left(I_{b}^{\mu }\left(i,j\right)\left|\frac{T_{70}\left(i,j\right)}{255}\right.\right),$$

where the quantization level of the JPEG compression was set to Q=70. The parameters of the QAF in (13) were set as gradient β=1000, and the number of the hyperbolic tangent functions n=500. For all the QAF-applied blocks, an IDCT was performed, and the luminance values were inversely normalized using (18). Next, all blocks were combined. The combined image was converted back to the original image size. Then, the QAF-applied image was given by

(22) $$I_{\mathrm{QAF}}=\left(I_{\mathrm{QAF}}^{1},I_{\mathrm{QAF}}^{2},\cdots ,I_{\mathrm{QAF}}^{4096}\right).$$

In general, the peak signal-to-noise ratio (PSNR) of an evaluated image $I^{\prime }$ against a reference image I is defined by

(23) $$\mathrm{PSNR}\left(I^{\prime }|I\right)=10\log_{10}\left(\frac{255^{2}}{\mathrm{MSE}\left(I^{\prime },I\right)}\right)\left[\mathrm{dB}\right],$$

where

(24) $$\mathrm{MSE}\left(I^{\prime },I\right)=\frac{1}{HW}\sum_{i=0}^{H-1}\sum_{j=0}^{W-1}{\left\{I^{\prime }\left(i,j\right)-I\left(i,j\right)\right\}}^{2}.$$

**Figure 9:**
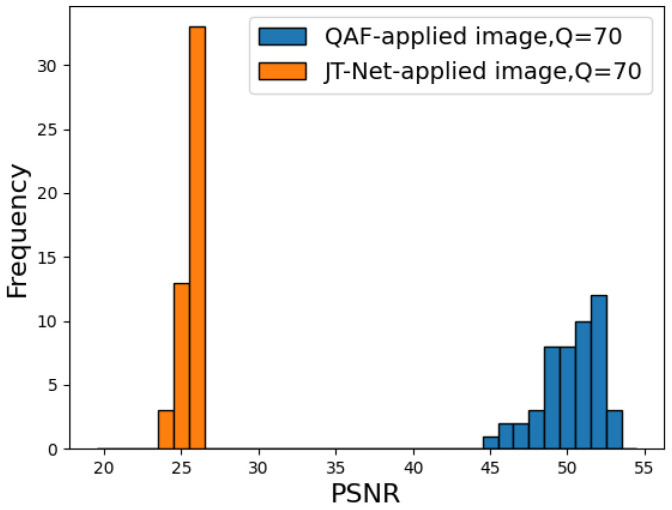
Histogram of PSNRs for JT-Net-applied images and QAF-applied images.

**Figure 10:**
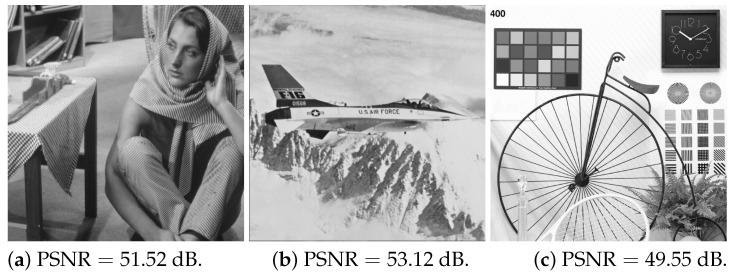
QAF-applied images and their PSNRs.

To see the difference between the QAF and JPEG-compressed images shown in Figure 6, the difference could be evaluated by PSNR rather than MSE. The accuracy of the QAF could be measured by the PSNR of a QAF-applied image against a JPEG-compressed image, that is, $\mathrm{PSNR}\left(I_{\mathrm{QAF}}|I_{\mathrm{jpeg}}\right)$, where $I_{\mathrm{jpeg}}$ is the JPEG-compressed image. Note that the PSNR was measured against the JPEG-compressed image, not the original image. Similarly, we evaluated the approximation ability of JT-Net using PSNR and compared it with QAF. Figure 9 shows a histogram of PSNRs for JT-Net-applied images by using (9) and QAF-applied images given by (21) and (22), where the quantization level is Q=70. The PSNRs for QAF-applied images are clearly greater than those for JT-Net-applied images. Figure 10 shows three examples of QAF-applied images and their PSNRs. Thus, we found that the QAF more adequately represents the quantization of the JPEG compression.

### 5.2. Evaluation of the Proposed Attack Layer

We compared the JT-Net in the ReDMark, our previous model, and the proposed quantization table-based QAF network (QT-QAF-Net) based on the image quality of stego-images and the BER of watermarks extracted from stego-images after the JPEG compression. Note that our previous model [15] is the AE-based model with the quantization table quantized by constant intensity. For comparison, we used an improved model of the CNN-based QAF network with a constant intensity quantization table (constant QAF-Net). The only difference between the QT-QAF-Net and the constant QAF-Net is the values of the quantization table.

#### 5.2.1. Experimental Conditions

The training and test images were selected as they were in ReDMark [11]. The training images were 50,000 images of 32×32 pixels from CIFAR10 [21] (H=32,W=32). An h×w-bit watermark was embedded, where h=4,w=4. The watermark was randomly generated. Therefore, the amount of watermark embedded per pixel was approximately 0.0156 bits per pixel. In the parameters used for training, the block height and width sizes were set to M=8 and N=8, respectively. The parameter of the loss function (20) was set to γ=0.75, the number of learning epochs was set to 100, and the mini-batch size was set to 32. For the parameters of SGD, the training rate was set to $10^{-4}$, and the moment was set to 0.98. For training the proposed attack layer, the gradient of the QAF (13) was set to β=1000, and the number of the hyperbolic tangent functions was set to n=500. In the attack layer of JT-Net and the proposed method, the quantization level was set to Q=70, and the quantization table $T_{70}$ was used. The embedding, attack, and extraction networks were all connected, and the network was trained using the training images and watermarks. Here, the embedding intensity was fixed at α=1.

For testing, 49 images of 512×512 pixels from the University of Granada were used. These images were divided into 32×32 pixel subimages, and the embedding process was performed on each of them. The 256 subimages were given to the network as test images. Meanwhile, a 32×32-bit watermark was randomly generated. One watermark was embedded four times in one image. That is, the watermark was divided into 4×4-bit subwatermarks, and finally each subwatermark was embedded in one subimage. An estimated watermark was determined by bit-by-bit majority voting because the same watermark was embedded four times in one image.

As stated in Section 3.1, the attack layer was not used during testing. The test images and the watermarks were used to output stego-images in the embedding network. Here, the embedding intensity was set to values from α=0.5 to 1.0. The stego-image is published. Subsequently, we assumed that it was JPEG-compressed by some JPEG tool with quantization levels Q=10,20,⋯,90. The compressed stego-images were input to the extraction network, and the estimated watermarks were output. These networks were trained 10 times with different initial weights for the comparison of our network with the JT-Net on image quality and BER. The mean and standard deviation of structural similarity index measures (SSIMs), PSNRs, and BERs were calculated.

#### 5.2.2. Evaluation of the Image Quality

**Figure 11:**
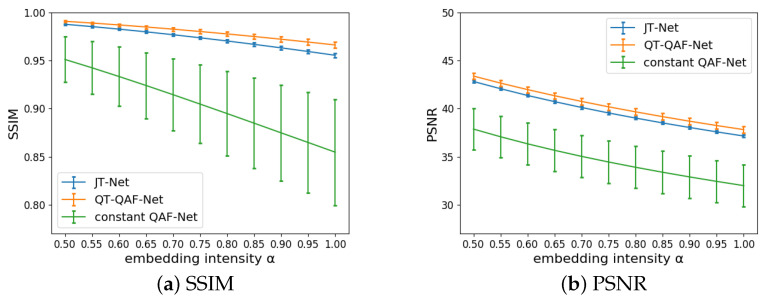
Image quality of stego-images (tained by Q=70).

The image quality of the stego-images obtained from the JT-Net, the constant QAF-Net and QT-QAF-Net was evaluated using the SSIMs and PSNRs. The image quality of the stego-image $I_{st}$ against the original image I can be expressed as $\mathrm{SSIM}\left(I_{st},I\right)$ by (17) and $\mathrm{PSNR}\left(I_{st}|I\right)$ by (23). Figure 11 shows the SSIM and PSNR. The horizontal and vertical axes represent the embedding intensity α and SSIM or PSNR, respectively. The error bars represent the standard deviation of the SSIMs and PSNRs. Embedding a watermark strongly causes degradation in image quality. Therefore, the SSIM and PSNR decreased as the intensity α increased. The image quality of the proposed QT-QAF-Net was higher than that of the other two networks. In other words, our network can reduce the degradation of image quality even with the same embedding intensity.

**Figure 12:**
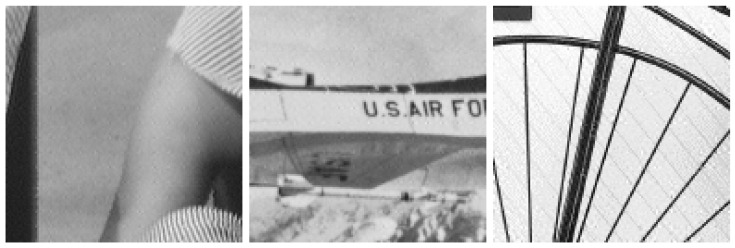
Images cropped to 128×128 pixels.

As the images were processed block by block, they were visually checked for block artifacts. Three images selected from the dataset were cropped to 128×128-pixel size as shown in Figure 12. These images were generated from the proposed network trained with embedding intensity α=1.0. The images were not reduced in size when displayed. Few noticeable artifacts were observed.

#### 5.2.3. Evaluation of the BER

The robustness of our network against the JPEG compression was evaluated. The estimated watermark obtained by (8) was evaluated by using the BER. The BER of the estimated watermark $p_{e}$ can be defined by

(25) $$\mathrm{BER}=\frac{1}{hw}\sum_{i=0}^{h-1}\sum_{j=0}^{w-1}p\left(i,j\right)⊕p_{e}\left(i,j\right),$$

where p is the original watermark, and ⊕ represents the exclusive OR.

**Figure 13:**
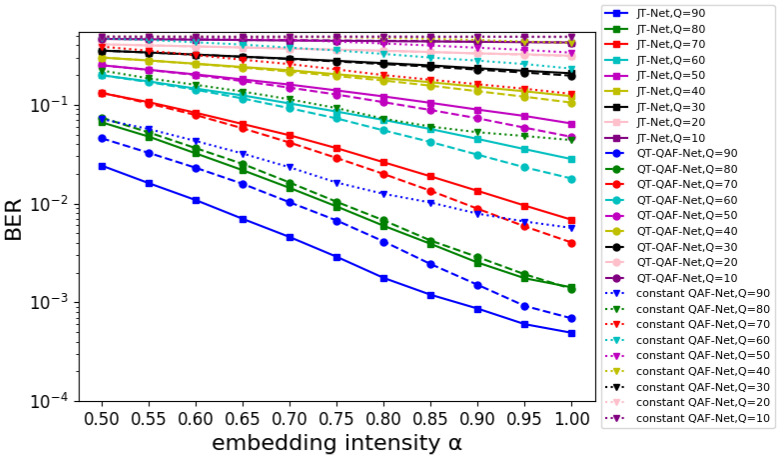
BER for embedding intensity α.

First, we compared the robustness of our network with that of the JT-Net and constant QAF-Net using the same embedding intensity α. Figure 13 shows the BER of the estimated watermark for the embedding intensity α. The horizontal and vertical axes represent the intensity α and BER, respectively. For different compression levels *Q*, the dashed lines with circles represent the BER for the QT-QAF-Net, the solid lines with squares represent the BER for the JT-Net, and the dotted lines with triangles represent the BER for the constant QAF-Net. When the watermark was strongly embedded, it was extracted correctly. Therefore, the BER decreased as the intensity α increased. The lowest BER was obtained when α=1.0. At compression level Q≤70, the BER of the proposed network was lower than that of the JT-Net. Also, at Q=80, they had almost the same BER. Furthermore, at Q=90, the BER of our network was larger than that of the JT-Net. The BER of the constant QAF-Net was always larger than that of the other two networks.

**Figure 14:**
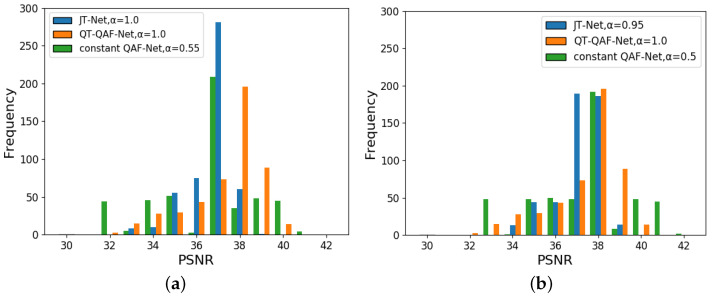
Histogram of PSNR for embedding intensity α=1.0 for QT-QAF-Net: (**a**) intensity α=1.0 for JT-Net and α=0.55 for constant QAF-Net and (**b**) intensity α=0.95 for JT-Net and intensity for constant QAF-Net α=0.50.

**Figure 15:**
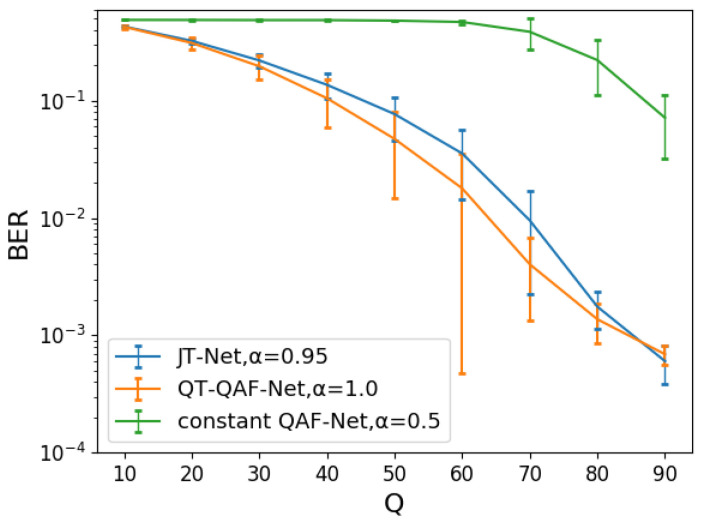
BER for the JPEG compression level *Q*.

Even with the same embedding intensity, the image quality of our network differs from that of the JT-Net and the constant QAF-Net. Therefore, we next adjusted the embedding intensity so that the PSNRs of these three networks were approximately the same, and we compared the BERs of the networks under this condition. Figure 14 shows the histograms of PSNRs for the QT-QAF-Net with embedding intensity α=1.0. Figure 14a shows the histograms for the embedding intensity α=1.0 for the JT-Net and α=0.55 for the constant QAF-Net, respectively. Figure 14b shows histograms for these embedding intensities α=0.95 and α=0.50, respectively. The intensities of the three networks were chosen so that the histograms shown look similar. To measure the robustness against JPEG compression, we set the intensity α to ensure that the PSNR obtained from these networks is approximately the same. Specifically, we set the intensity for the JT-Net and QT-QAF-Net to α=0.95 (average PSNR =37.58 dB) and α=1.0 (average PSNR =37.81 dB), respectively. Figure 15 is the BER for the compression level *Q*. The error bars are the standard deviation of the BERs. The BER of the estimated watermark for QT-QAF-Net is lower than that for the JT-Net and constant QAF-Net. Thus, we can say that our network can generate watermarks with fewer errors under the given PSNR.

## 6. Conclusions

The JT-Net in ReDMark [11] is a network that simulates JPEG compression. This network substitutes the quantization process with a process that adds noise proportional to the value of the quantization table. In previous work [15], a quantized activation function (QAF) quantized by constant intensity (constant QAF-Net) was proposed. In this paper, we proposed the quantization table-based QAF network (QT-QAF-Net), which can approximate the quantization process of the JPEG compression according to the standard. By approximating the quantization of the JPEG compression using the QAF, we expected to improve the robustness against the JPEG compression. The results of computer simulations showed that the QAF represented quantization with sufficient accuracy. Also, we found that the network trained with the QAF was more robust against the JPEG compression than those trained with the JT-Net. Because the embedding and extraction networks were more robust against the JPEG compression when trained with the QAF, we conclude that our method is more suitable for simulating JPEG compression than conventional methods applying additive noise.

Further studies with QAF are expected. For example, since QAF is differentiable over the whole interval, it may produce better adversarial images compared to JPEGdiff [12]. Furthermore, there is a study on the estimation of the sign bit of DCT coefficients [22]. The non-linearity of the quantized DCT coefficients makes estimation difficult. The solution could be simplified by using QAF.

## Data Availability

Publicly available datasets were analyzed in this study. These data can be found here: CIFAR-10 dataset https://www.cs.toronto.edu/~kriz/cifar.html (accessed on 3 June 2024) and Computer Vision Group. University of Granada https://ccia.ugr.es/cvg/CG/base.htm (accessed on 3 June 2024).
